# Validity of Heart Failure Diagnoses in Administrative Databases: A Systematic Review and Meta-Analysis

**DOI:** 10.1371/journal.pone.0104519

**Published:** 2014-08-15

**Authors:** Natalie McCormick, Diane Lacaille, Vidula Bhole, J. Antonio Avina-Zubieta

**Affiliations:** 1 Faculty of Pharmaceutical Sciences, University of British Columbia, Vancouver, British Columbia, Canada; 2 Arthritis Research Centre of Canada, Richmond, British Columbia, Canada; 3 Division of Rheumatology, Department of Medicine, University of British Columbia, Vancouver, British Columbia, Canada; 4 Cardiovascular Committee of the Canadian Rheumatology Administrative Data Network, Richmond, British Columbia, Canada; 5 EpiSolutions Consultancy Services, Thane, India; University of Louisville, United States of America

## Abstract

**Objective:**

Heart failure (HF) is an important covariate and outcome in studies of elderly populations and cardiovascular disease cohorts, among others. Administrative data is increasingly being used for long-term clinical research in these populations. We aimed to conduct the first systematic review and meta-analysis of studies reporting on the validity of diagnostic codes for identifying HF in administrative data.

**Methods:**

MEDLINE and EMBASE were searched (inception to November 2010) for studies: (a) Using administrative data to identify HF; or (b) Evaluating the validity of HF codes in administrative data; and (c) Reporting validation statistics (sensitivity, specificity, positive predictive value [PPV], negative predictive value, or Kappa scores) for HF, or data sufficient for their calculation. Additional articles were located by hand search (up to February 2011) of original papers. Data were extracted by two independent reviewers; article quality was assessed using the Quality Assessment of Diagnostic Accuracy Studies tool. Using a random-effects model, pooled sensitivity and specificity values were produced, along with estimates of the positive (LR+) and negative (LR−) likelihood ratios, and diagnostic odds ratios (DOR = LR+/LR−) of HF codes.

**Results:**

Nineteen studies published from1999–2009 were included in the qualitative review. Specificity was ≥95% in all studies and PPV was ≥87% in the majority, but sensitivity was lower (≥69% in ≥50% of studies). In a meta-analysis of the 11 studies reporting sensitivity and specificity values, the pooled sensitivity was 75.3% (95% CI: 74.7–75.9) and specificity was 96.8% (95% CI: 96.8–96.9). The pooled LR+ was 51.9 (20.5–131.6), the LR− was 0.27 (0.20–0.37), and the DOR was 186.5 (96.8–359.2).

**Conclusions:**

While most HF diagnoses in administrative databases do correspond to true HF cases, about one-quarter of HF cases are not captured. The use of broader search parameters, along with laboratory and prescription medication data, may help identify more cases.

## Introduction

Heart failure (HF) is a chronic condition that affects about 26 million people worldwide [1] and imposes a tremendous burden on these individuals and their families. The typical 40 year-old faces a 20% lifetime risk of developing HF [2], and the incidence of HF amongst adults 65 years of age and older is approximately 12.5 per 1,000 person-years [3]. About half of new cases are expected to die within five years of diagnosis [2], and estimates of the annual economic burden of HF have recently exceeded $30 billion in the United States [2], and $108 billion worldwide [4].

The European Society of Cardiology describes HF as a disorder of cardiac structure or function where the heart is unable to deliver adequate levels of oxygen to the tissues [5]. Cases often have primary left systolic HF, which is characterized by “reduced contraction and emptying of the left ventricle” [5]. Still, many cases have left diastolic HF, in which ventricular compliance and filling are impaired [6] but the contractile function of the ventricle is preserved. HF has some ‘classic’ signs and symptoms, including ankle oedema, and exertional dyspnoea and fatigue [5], [6]. However, HF is not considered to be a discrete condition but a “complex clinical syndrome” [6] that occurs in conjunction with other cardiovascular diseases such as coronary artery disease, valvular heart disease, hypertension, dilated cardiomyopathy [6], and conduction and rhythm disorders [5]. A significant source of morbidity on its own, HF frequently occurs in concordance with other chronic disorders such as renal disease [7]–[9], chronic obstructive pulmonary disease (COPD) [8], [10]–[12], and diabetes [7]–[11]. Thus when evaluating treatments for these and other chronic conditions, it is essential to adjust for diagnoses of HF.

Administrative databases have become excellent resources for the study of HF by allowing for long-term evaluation of large numbers of patients at relatively low cost. Some examples are the Medicare databases in the United States (USA) and health ministry databases from countries such as Canada where healthcare is funded by provincial governments and available to all residents. These data sources allow the patient-level linkage of health resource utilization data (including hospital separations, outpatient encounters, and sometimes, dispensed prescriptions) to demographic and vital statistics data. When studying clinic-based populations, patients with severe HF are likely to be overrepresented, but administrative databases provide a means for identifying risk factors for HF, and quantifying the effects of treatment in unselected populations.

However, administrative databases are only useful for HF research if the diagnostic codes contained within are valid; that is, if they can be used to distinguish those who actually have HF from those who do not. Their validity can be assessed by comparing the administrative database diagnosis to an accepted ‘gold standard’ reference diagnosis. This diagnosis is typically obtained through more resource-intensive processes such as patient self-report, retrospective chart review, or prospective clinical examination. Principal measures of validity include sensitivity (how many HF cases in the population are actually coded for HF) and specificity (how many of the non-HF cases in the population are, in turn, *not* coded for HF). Unfortunately, there is some uncertainty surrounding the validity of diagnoses recorded in administrative databases since most databases are not established for research purposes. Validity is of particular concern when studying HF patients, as they tend to have high comorbidity burdens and be hospitalized for other cardiovascular and respiratory conditions [13], [14]. While HF may have contributed to the need for these hospitalizations, this diagnosis may not be entered on the discharge record, leaving this potential confounding variable to go undetected in subsequent epidemiologic investigations. Although several assessments of the validity of HF codes in administrative databases have been published [15]–[17], there is considerable heterogeneity amongst them with regards to the clinical settings and reference standards used. Of note, many of these assessments were limited to specific populations (e.g. those diagnosed with atrial fibrillation [15] or myocardial infarction (MI) [16]) so may not be generalizable to the HF diagnoses recorded for other individuals.

As a part of a Canadian Rheumatology Network for establishing best practices in the use of administrative data for health research and surveillance (CANRAD) [18]–[22], we have conducted a systematic review of studies reporting on the validity of diagnostic codes for identifying cardiovascular diseases (CVD) in administrative data. Data from these studies were used to compare the validity of these codes, and to evaluate whether administrative health data can accurately identify CVD for the purpose of identifying these events as covariates, outcomes, or complications in future research. We recently reported our findings on the validity of codes for MI [23]. In the current paper, we focus on HF and undertake both a qualitative analysis, and for the first time, a quantitative synthesis of studies reporting on the validity of HF codes in administrative databases.

## Methods

### Literature Search

Comprehensive searches of the MEDLINE and EMBASE databases from inception (1946 and 1974, respectively) to November 2010 for all available peer-reviewed literature were conducted by an experienced librarian (M-DW). Two search strategies were employed: (1) All studies where administrative data was used to identify CVD; (2) All studies reporting on the validity of administrative data for identifying CVD. Our MEDLINE and EMBASE search strategies are available as (Text S1 and S2). To find additional articles, the authors hand-searched the reference lists of the key articles located through the database search. The Cited-By tools in PubMed and Google Scholar were also used to find relevant articles that had cited the articles located through the database search (up to February 2011). The titles and abstracts of each record were screened for relevance by two independent reviewers. No protocol for this systematic review has been published, though the review was conducted in accordance with the Preferred Reporting Items for Systematic Reviews and Meta-Analyses (PRISMA) [24] and Meta-Analysis of Observational Studies in Epidemiology (MOOSE) [25] statements; our completed checklists are provided as (Checklist S1 and S2).

### Inclusion Criteria

We selected full-length, peer-reviewed articles published in English that used administrative data and reported validation statistics for the main International Classification of Diseases (ICD) codes for HF (ICD-8 and ICD-9 428, and ICD-10 I50), or provided sufficient data enabling us to calculate them. Any discrepancies were discussed until consensus was reached. When the conflict persisted a third reviewer (JAA-Z) was consulted.

### Data Extraction

The full text of each selected record was examined by two independent reviewers (NM and VB) who abstracted data using a standardized collection form (a copy is provided in **Text S3**). While extracting data, particular attention was given to the study population, administrative data source, algorithm used to identify HF, validation method, and gold standard. Validation statistics comparing the HF codes to definite or possible cases were abstracted. These statistics included sensitivity, specificity, positive predictive value (PPV), negative predictive value (NPV), and kappa scores. Because hospital separations typically contain multiple diagnoses, with the primary or principal diagnosis in the first position followed by one or more secondary diagnoses, we abstracted statistics for each of these positions, where available. This was especially important given some recent studies of administrative databases that suggest hospitalizations with HF in the primary position are decreasing, while those with HF coded in secondary diagnostic positions are increasing [26],[27]. Data were independently abstracted by each reviewer who subsequently compared their forms to correct any errors and resolve discrepancies.

### Quality Assessment

The design and methods used by each study, including the rigour of the reference standard, can directly influence the validity statistics produced. Thus, all studies were evaluated for quality, and the validation statistics were stratified by level of study quality. This was done using the Quality Assessment of Diagnostic Accuracy Studies (QUADAS) tool [28] (available as a part of **Text S3**), used previously by the CANRAD network in assessing the validity of codes for diabetes mellitus [21], osteoporosis and fractures [22], and myocardial infarction [23]. Briefly, it is a 14-item evidence-based quality assessment tool used in systematic reviews of diagnostic accuracy studies. Each item, phrased as a question, addresses one or more aspects of bias or applicability; however, there is no overall score. Instead, as done previously [22], [23], items were independently answered by each reviewer and used to qualitatively assess each study as High, Medium, or Low quality. Any disagreements were resolved by consensus.

### Statistical Analysis

All validation statistics were abstracted as reported. Where sufficient data were available we calculated 95% confidence intervals (95% CI) and additional validity statistics not directly reported in the original publication. These were evaluated on aggregate, and, as pre-specified, stratified by geographic region and time period of publication. In evaluating the HF codes in administrative data, we considered the diagnosis assigned during the validation process to be the diagnostic gold standard; this meant, for instance, that cases coded for HF and classified as HF during validation were true-positive cases, while cases coded for HF but classified during validation as no-HF were false-positives. Sensitivity (the ability of the codes to identify true positive HF cases) was equal to the number of true positives divided by the sum of true positives and false negatives (all those with HF). Specificity (the ability of the HF codes to exclude false-positive cases) was equal to the number of true negatives divided by the sum of true negatives and false positives (all those without HF). Sensitivity and specificity were also used to calculate the positive and negative likelihood ratios (LR+ and LR−) and diagnostic odds ratios (DOR). The DOR (the ratio of the odds that coded individuals will actually have HF compared to the odds that non-coded individuals will have HF) was equal to the LR+ divided by the LR−.

The positive likelihood ratio (LR+), the ratio of true-positives to false-positives amongst all those coded for HF, was equal to the sensitivity divided by 1 – specificity. The negative likelihood ratio (LR−), the ratio of false-negatives to true-negatives amongst all those not coded for HF, was equal to 1 – sensitivity divided by the specificity. Thus, higher LR+ values (those greater than 1) mean the presence of an HF code is more indicative of true HF and lower LR- values (those closer to 0 than 1) mean the absence of an HF code is more indicative of non-disease (no HF). Specificity values typically fall close to 1, such that the denominator for LR+ (1 – specificity) is usually much smaller than the denominator for LR−. As a result, the values for LR+ (which range from 1 to 10 or more) are usually much larger than those for LR− (which range from 0 to 1). An LR+ of 5 to 10 means the codes are moderately good for detecting HF, and an LR+>10 means the codes are very good. Similarly, with an LR− of 0.1 to 0.2, the absence of an HF code corresponds moderately to non-disease (no HF), while an LR− of <0.1 corresponds very well to non-disease [29].

Three other validation statistics of interest were PPV, NPV, and kappa score. The PPV (the likelihood that the HF code corresponds to a true-positive case) was equal to the number of true positives divided by the total number of cases coded for HF (true-positives and false-positives). NPV (the likelihood that an individual not coded for HF is a true-negative case) was equal to the number of true negatives divided by the total number of cases not coded for HF (true-negatives and false-negatives). Kappa (a measure of the agreement, beyond that expected by chance, between how cases are classified in the administrative database and by the validation process) was equal to the observed agreement (the percent of cases classified as either true-positives or true-negatives) minus that expected by chance, divided by [100% - the agreement expected by chance]. Kappa scores greater than 0.60 indicate substantial/almost perfect agreement, 0.41–0.60 is considered as moderate agreement, 0.21–0.40 as fair agreement, and those 0.20 or lower as light/poor agreement [30].

Where available, we abstracted statistics for definite and possible cases of HF, though the number of categories reported depended on the choice of reference standard. In some studies, the reference standard is the presence of any notation of an HF diagnosis in the medical chart, and cases are classified simply as HF or no HF. The Framingham criteria [31] also classify cases as either HF or no HF; at least two of the major Framingham criteria (which include neck vein distension, cardiomegaly, and acute pulmonary edema) or one major criterion and two minor criteria (which include ankle oedema, hepatomegaly, and plural effusion) must be met for the diagnosis of HF. Other sets of standard criteria do allow for further classification. The Carlson criteria [32] use a points system in which potential cases are evaluated in three categories (history, physical examination, and chest radiography), and allocated a maximum of four points in each category, and a maximum overall score of 12. A score of 8 or more is considered Definite HF while 5–7 points are considered Possible HF, and 4 or fewer points are classified as Unlikely HF [32]. Under the European Society of Cardiology criteria [33], for a case to be classified as HF there must be both signs and symptoms of HF, and objective evidence of cardiac dysfunction. Some investigators [17], [34] have classified cases meeting both of these criteria as Definite HF, and those meeting only one of these criteria as Questionable, Possible, or Probable HF. It should be noted that while the New York Heart Association functional classification is used to measure the degree of functional limitation experienced by HF patients, and may assist in the selection of therapies [5], it is not used to make the initial diagnosis of HF.

### Meta-Analysis

Studies that reported raw data for sensitivity and specificity were included in the meta-analysis. Forest plots and a summary receiver operating characteristic (ROC) curve were constructed, and pooled estimates (and 95% CI's) of the sensitivity and specificity values, LR+, LR−, and DORs were calculated. More informative diagnostic tests (in this case, being HF codes) - those with good sensitivity and good specificity - will produce ROC curves positioned in the top-left area of the ROC plane, well away from the positive diagonal line [35]. Two additional summary measures of test performance were determined from the ROC curve, the area under the curve (AUC) and Q*. The AUC ranges between 0 and 1, with 1 corresponding to a perfect test [36]. In the context of our research question, an AUC of 1 would mean that, given two cases, one with HF and one without, there is a 100% probability that the positive case will be coded for HF and the negative case will not. The Q*, the lower bound of the AUC, is the point at which the sensitivity and specificity are equal [36]. Higher Q* values indicate better-performing tests.

To assess for the presence of heterogeneity amongst the included studies, we visually inspected the forest plots and ROC curve, and calculated the χ^2^ statistic, Cochran's Q [37] and I^2^ [38] statistics. The I^2^ index, a measure of the degree of inconsistency across study findings, is expressed as the percentage of variation between studies due to heterogeneity as opposed to chance [38]. A value of 0% indicates no observed heterogeneity, while 25% is indicative of low heterogeneity, 50% moderate, and 75% high heterogeneity [38]. When there are a small number of studies, the I^2^ index is a preferred measure over Cochrane's Q [38]. In the absence of substantial heterogeneity, a fixed-effects model was to be applied. Otherwise, a random-effects model was to be applied, using the DerSimonian Laird method.

To assess the impact each individual study had on the pooled estimates, a jackknife sensitivity analysis [39] was performed in which one study was removed and all summary statistics were re-calculated. This process was repeated for all studies. The impact of publication bias was not evaluated as the common tests available to assess publication bias, including the Begg, Egger, and Macaskill tests, have been shown to be misleading for meta-analyses of test accuracy [40]. All analyses were conducted using Meta-Disc software, version 1.4 [41].

## Results

### Literature Search

**Figure 1:**
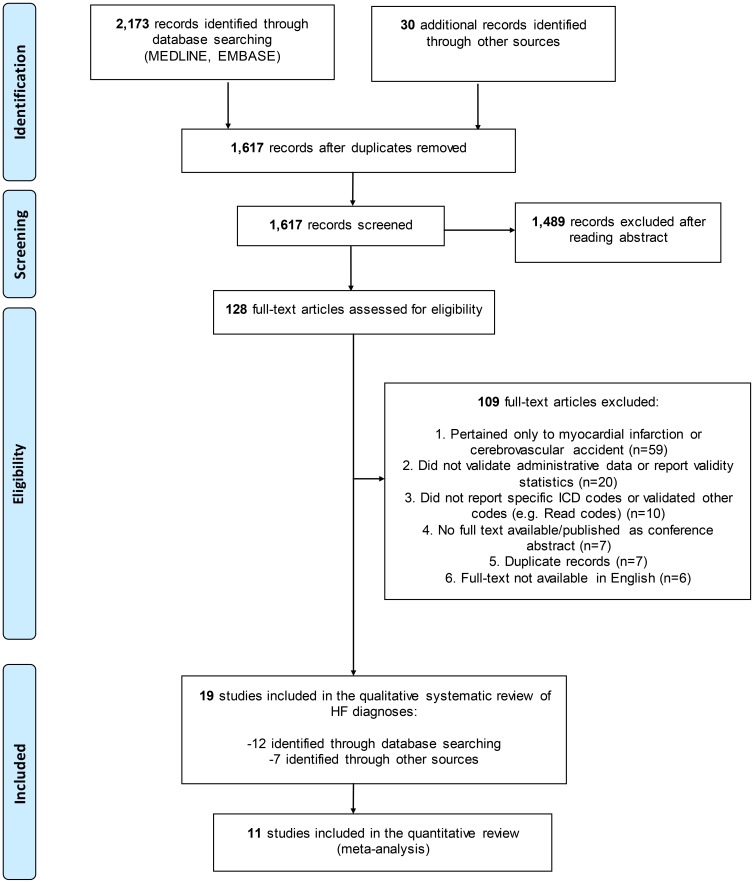
Preferred Reporting Items for Systematic Reviews and Meta-Analyses (PRISMA)-style Flowchart of Study Selection and Review. HF = heart failure; ICD = International Classification of Diseases

After the removal of duplicates, 1,587 citations were identified through MEDLINE and EMBASE searches and screened for relevance to our study objectives. We then assessed 98 full-text articles for eligibility (**Figure 1**), of which 12 were selected for inclusion. We also assessed 30 full-text articles for eligibility that were identified from hand searches, and selected 7 additional articles therein. Thus, a total of 128 articles were assessed for eligibility, from which 109 were excluded, mainly because they reported on the validity of other CVD (n = 59), or did not actually validate HF diagnoses in administrative data (n = 20). Six articles were excluded because they were not published in English; their languages of publication were Danish, German, Italian, Japanese, Portuguese, and Spanish. Ultimately 19 articles were included for the qualitative systematic review of HF.

### Study Characteristics

Of the 19 articles evaluating HF diagnoses that were included in the final review, nine (47%) were from the United States, six (32%) were from Canada, three (16%) from Europe, and one was from Australia. Characteristics of these studies are presented in **Table 1**. Validation was the primary objective in all but one [42] of these studies. Six studies [17], [34], [42]–[45] reported on the validity of HF exclusively, while 13 reported on the validity of other diagnoses as well. Overall, data were collected over a thirty-year period (1976–2005), though the studies were all published relatively recently (the earliest in 1999 [16]). PPV data were available from all but one [16] study, sensitivity and NPV data were available from 14 studies (74%), and specificity data from 13 studies (68%). Kappa scores were less frequently reported. Only four studies [17], [46]–[48] reported on the validity of ICD-10 codes separately from ICD-9. Most of the administrative databases pertained to hospitalizations though diagnoses recorded for outpatient encounters were included in five studies [42], [44], [45], [49], [50]. None of the studies reported on the validity of HF as a cause-of-death.

**Table 1:** Characteristics of Included Studies.

First Author, Year of Publication	Year(s) of Data Collection	Primary Validation Study?	Country	Records Evaluated (N)	Source Population	Type of Administrative Data	Gold Standard
**Alqaisi** [45], 2009	2004–2005	Yes	USA (Michigan)	400	enrollees in large HMO receiving care from large multi-specialty medical group	ICD-9 inpatient/outpatient records	CRDC – Framingham criteria
**Austin** [53], 2002	1996–2000	Yes	Canada (Ontario)	58,816	consecutive patients ≥20 years old admitted to coronary care units from emergency room	ICD-9 inpatient records	disease registry - diagnosis of bedside nurse (as recorded in Fastrak II prospective acute coronary syndromes registry)
**Birman-Deych** [15], 2005	1998–1999	Yes	USA	23,657	Medicare beneficiaries (aged 20-105 years) hospitalized for atrial fibrillation	ICD-9 inpatient records	chart review
**Chen** [47], 2009	2003	Yes	Canada (Alberta)	4,008	general hospitalized population	ICD-10 inpatient records	chart review
**Goff** [43], 2000	1988–1994	Yes	USA (Texas)	5,083	general hospitalized population (participants in population-based Corpus Christi Heart Project, all residents 25-74 years of one Texas county)	ICD-9 inpatient records	chart review
**Heckbert** [55], 2004	1994–2000	Yes	USA	34,016	women participating in the Women's Health Initiative clinical and observational studies	ICD-9 inpatient records	CRDC – Women's Health Initiative criteria
**Heisler** [52], 2009	2004–2005	Yes	USA (Minnesota)	712	women undergoing vaginal hysterectomy for benign indication	ICD-9 inpatient records	chart review
**Henderson** [46], 2006	1998–1999, 2000–2001	yes	Australia	14,635	all hospitalized patients (excluding same-day chemotherapy and dialysis)	ICD-10 inpatient records	chart review: charts were re-coded by professional coders
**Humphries** [51], 2000	1994–1995	yes	Canada (British Columbia)	817	patients hospitalized for percutaneous coronary intervention	ICD-9 inpatient records	chart review
**Ingelsson** [34], 2005	1976–2001	yes	Sweden	317	men (aged 50 years at enrolment) in a cohort study aimed at identifying metabolic risk factors for cardiovascular disease	ICD-8,9,10 inpatient records	CRDC - European Society of Cardiology criteria
**Khand** [17], 2005	1997–1998	yes	UK	339	hospitalized patients	ICD-10 inpatient records	CRDC - European Society of Cardiology criteria
**Lee** [56], 2005	1997–1999	yes	Canada (Ontario)	1,808	general hospitalized population (<105 years)	ICD-9 inpatient records	CRDC - Framingham and Carlson criteria
**Levy** [16], 1999	1994	yes	Canada	224	individuals ≥65 years hospitalized with myocardial infarction	ICD-9 inpatient records	chart review: mentioned in records
**Merry** [54], 2009	1987–2003	yes	Netherlands	21,110	residents of one region of the Netherlands, aged 20–59 years at registration, participating in two large monitoring projects	ICD-9 inpatient records	disease registry - diagnosis listed in Cardiology Information System (hospital cardiology service)
**Onofrei** [44], 2004	1998–2000	yes	USA (Oregon)	205,756	all patients attending a network of nine internal medicine and family practice clinics	ICD-9 inpatient/outpatient records	CRDC - left ventricular ejection fraction value
**Rector** [49], 2004	1999–2000	yes	USA (Midwest and Northeast)	3,633	Medicare beneficiaries ≥65 years of age	ICD-9 inpatient/outpatient records	patient self-report
**Roger** [42], 2004	1979–2000	no	USA (Minnesota)	7,298	population-based heart failure cohort	ICD-9 inpatient/outpatient records	CRDC - Framingham criteria, and chart review – mentioned in chart
**So** [48], 2006	2003	yes	Canada (Alberta)	193	patients hospitalized for myocardial infarction	ICD-9,10 inpatient records	chart review
**Szeto** [50], 2002	1996–1998	yes	USA (California)	148	patients attending a Veterans Affairs general medicine clinic	ICD-9 outpatient records	chart review - mentioned in records/problem list

CRDC = Chart Review, Diagnostic Criteria – the charts of potential cases were reviewed, and a formal set of diagnostic criteria were applied when evaluating cases.

Chart reviews, sometimes in conjunction with unspecified diagnostic criteria, formed the basis of the gold standard in nine (47%) studies [15], [16], [43], [46]–[48], [50]–[52], and patient self-report was used in one [49]. Cardiac disease registries were used in two studies [53], [54], while a specific set of diagnostic criteria were incorporated in the reference standards of the seven remaining studies [17], [34], [42], [44], [45], [55], [56].

Study quality was evaluated based on the QUADAS tool [28], with 11 of 19 studies (58%) categorized as high quality, seven as medium (37%), and just one (5%) as low quality. A detailed breakdown of the quality assessment for each study is provided in **Table S1**. Of the seven medium-quality studies, two did not adequately describe the validation process [47], [48], and five used a less-reliable gold standard than published diagnostic criteria (being patient self-report in one [49] and chart review by an individual other than a clinician or trained hospital coder in four [15], [16], [50], [51]). The low-quality study [52] employed a very select source population (women at one institution undergoing vaginal hysterectomy), assessed only two potential cases of HF, and did not adequately describe the validation process.

### Validity of Heart Failure Diagnoses

The validation statistics reported by each of the included studies are provided in **Table 2**. Sensitivity was reported by 14 studies, and was ≥69% in half of them (range: 0 to 87%). PPV was undefined (0/0) in one of the studies [52], but was at least 87% in nine of the 17 remaining studies (range: 34 to 100%). Specificity was ≥95% in all 13 studies reporting this statistic, and NPV was ≥88% in all but two of the 14 studies where this data was available. Kappa was only reported in six (32%) studies [43], [46], [47], [51], [53], [55]. The values in three of the studies (which ranged from 0.43 to 0.58) indicated there was moderate agreement between the diagnostic codes and reference standard, while those in the other three (range 0.72 to 0.94) indicated there was substantial to almost perfect agreement.

**Table 2:** Results of Studies Validating Diagnoses of Heart Failure (HF) in Administrative Data.

First Author, Year	Diagnostic Codes	Parameter	Sensitivity (95% CI)	Specificity (95% CI)	PPV (95% CI)	NPV (95% CI)	Kappa (95% CI)	LR+	LR-	DOR	Quality
**Levy** [16], 1999	ICD-9 402, 408, 425, 428, 429.3	HF as a comorbidity (secondary diagnosis) of MI	65 (59–72)			78 (72–83)		5.91 (3.47–10.05)	0.39 (0.30-0.51)	15.06 (7.47-30.37)	Medium
**Goff** [43], 2000	ICD-9 428	primary or secondary diagnosis	62.79 (60.17–65.34)	95.39 (94.65–96.03)	83.48 (81.04–85.66)	87.38 (86.28–88.35)		13.61 (11.69–15.85	0.39 (0.36-0.42)	34.90 (28.90-42.13)	High
	ICD-9 428 or 402	primary or secondary diagnosis	66.21 (63.63–68.69)	93.28 (92.42–94.06)	78.53 (76.04–80.84)	88.18 (87.09–89.19)		9.86 (8.69–11.18)	0.36 (0.34-0.39)	27.21 (22.95-32.26)	
	ICD-9 398.91, 402.x1, 404.x, 415.0, 416.9, 425.4, 428.x, 429.4, 514, 518.4, 786.0	primary or secondary diagnosis	67.08 (64.51–69.55)	92.61 (91.71–93.42)	77.11 (74.60–79.44)	88.34 (87.28–89.33)	0.72	9.08 (8.05–10.23)	0.36 (0.33-0.38)	25.53 (21.61-30.16)	
	ICD-9 428.x	females	66	95.3	86.3	86.2					
	ICD-9 428.x	males	60.5	95.7	81.9	88.2					
	ICD-9 428.x or 402.x	females	68.8	92.2	79.7	86.8					
	ICD-9 428.x or 402.x	males	64.6	93.2	75.5	89.0					
	ICD-9 398.91, 402.x1, 404.x, 415.0, 416.9, 425.4, 428.x, 429.4, 514, 518.4, 786.0	females	70.2	92.1	79.9	87.3					
	ICD-9 398.91, 402.x1, 404.x, 415.0, 416.9, 425.4, 428.x, 429.4, 514, 518.4, 786.0	males	65.0	93.2	75.5	89.1					
**Humphries** [51], 2000	ICD-9 428	as pre-admission comorbid diagnosis	66.1	94.8	50.6	97.2	0.53	12.71	0.36	35.28	Medium
**Austin** a [53], 2002	ICD-9 428	primary diagnosis	58.52 (57.20–59.83)	96.78 (96.63–96.93)	65.14 (63.78–66.46)	95.79 (95.61–95.95)	0.58	18.20 (17.29–19.17)	0.43 (0.42-0.44)	42.47 (39.51-45.65)	High
		primary or secondary	85.41 (84.44–86.33)	84.28 (83.97–84.59)	35.80 (34.98–36.63)	98.25 (98.13–98.37)	0.43	5.43 (5.31–5.56)	0.17 (0.16–0.19)	31.38 (29.01–33.94)	
**Szeto** [50], 2002	ICD-9 428.1, 428.0, 428.9	in administrative database	86.67 (58.39–97.66)	100.00 (96.50–100.00)	100.00 (71.66–100)	98.00 (94.21–99.74)		223.16 (14.1–3625.5)	0.16 (0.05–0.49)	1441.8 (65.77–31608.6)	Medium
**Onofrei** [50], 2004	ICD-9 402.01, 402.11, 402.91, 404.01, 404.03, 404.11, 404.13, 404.91, 404.93, 425.xx, 428.xx	HF defined as LVEF≤55%	43.9 (41.3–46.6)	99.5 (99.4–99.5)	35.6 (33.3–37.9)	99.6 (99.6–99.6)		80.40 (73.98–87.37)	0.56 (0.54–0.59)	142.54 (126.33–160.84)	High
		HF defined as LVEF≤40%	54.4 (50.8–57.9)	99.4 (99.3–99.4)	24.9 (22.9–27.0)	99.8 (99.8–99.8)		85.69 (78.81–93.17)	0.46 (0.43–0.50)	186.52 (160.54–216.71)	
**Heckbert** [55], 2004	ICD-9 428 or 425		78.99 (75.96–81.74)	97.72 (97.55–97.88)	45.34 (42.70–48.01)	99.49 (99.40–99.56)	0.56 (0.53–0.59)	34.67 (32.03–37.52)	0.22 (0.19–0.25)	161.27 (134.00–194.09)	High
	ICD-9 428 only				48.59 (45.78–51.41)						
	ICD-9 425 only				17.36 (11.75–24.76)						
**Rector** b [49], 2004	ICD-9 398.91, 402.01, 402.11, 402.91, 404.01, 404.11, 404.91, 404.03, 404.13, 404.93, 428.xx	from 1 year of data, diagnosis on 1 claim	57.80 (50.93–64.38)	92.83 (91.90–93.66)	33.96 (29.20–39.06)	97.18 (96.54–97.71)		8.06 (6.83–9.51)	0.46 (0.39–0.53)	17.72 (13.15–23.89)	Medium
		from 1 year of data, diagnosis on ≥2 claims	46.79 (40.06–53.64)	94.67 (93.85–95.39)	35.92 (30.39–41.83)	96.54 (95.85–97.12)		8.78 (7.19–10.72)	0.56 (0.50–0.64)	15.62 (11.51–21.19)	
**Roger** [42], 2004	ICD-9 428, as primary diagnosis	against Framingham criteria			82 (81.09–82.87)						High
		against clinical diagnosis			90 (89.28–90.67)						
**Khand** [17], 2005	ICD-10 I50.1, I50.2, I11.0, I11.1, I25.5, I42.9	possible, probable, or definite; 1^st^-6^th^ diagnostic positions			86.67 (82.41–90.05)						High
		probable or definite; 1^st^-6^th^ diagnostic positions			77.27 (72.29–81.60)						
		definite; 1^st^-6^th^ diagnostic positions			65.45 (60.02–70.53)						
**Ingelsson** [34], 2005	ICD-8 427.00, 427.10, 428.99; ICD-9 428; ICD-10 I50	overall definite			81.70 (76.91–85.71)						High
		overall definite or questionable			97.79 (95.31–99.03)						
		definite as primary diagnosis			95.00 (89.58–97.79)						
		definite or questionable as primary diagnosis			99.29 (95.49–99.96)						
**Lee** [56], 2005	ICD-9 428.x	vs. Framingham criteria, entire cohort; primary diagnosis			94.33 (93.07–95.38)						High
		vs. Framingham criteria, women; primary diagnosis			94.62 (92.81–96.01)						
		vs. Framingham criteria, men; primary diagnosis			93.91 (91.97–95.42)						
		vs. Carlson criteria, entire cohort; primary diagnosis			88.60 (86.94–90.08)						
		vs. Carlson criteria, women; primary diagnosis			89.35 (87.02–91.32)						
		vs. Carlson criteria, men; primary diagnosis			87.83 (85.32–89.96)						
**Birman-Deych** [15], 2005	ICD-9 428.x, 398.91, 402.01, 402.11, 402.91, 404.01, 404.11, 404.91, 404.03, 404.13, 404.93	any diagnostic position (current or past event)	76.00 (75.19–76.80)	97.95 (97.69–98.19)	97.00 (96.61–97.34)	82.41 (81.79–83.02)		37.10 (32.87–41.87)	0.25 (0.24–0.25)	151.44 (132.90–172.57)	Medium
		primary position, during baseline hospitalization (current event)	33.00 (32.13–33.89)	99.41 (99.26–99.53)	97.98 (97.46–98.40)	63.15 (62.47–63.81)		55.97 (44.59–70.24)	0.67 (0.67–0.68)	83.04 (65.95–104.56)	
		any position, during baseline hospitalization (current event)	83.00 (82.29–83.70)	86.00 (85.38–86.60)	85.00						
		any position, all hospitalizations within past 12 months of baseline (current event)	86.00 (85.33–86.64)	82.01 (81.33–82.68)	80.64 (79.91–81.35)	87.05 (86.43–87.65)		4.78 (4.60–4.97)	0.17 (0.16–0.18)	28.01 (26.11–30.05)	
**So** [48], 2006	ICD-9 428.x	as a comorbidity of MI	81.8 (68.65–90.48)	96.4 (91.75–98.81)	90.0 (78.19–96.67)	93.0 (87.52–96.60)		22.58 (9.47–53.87)	0.19 (0.11–0.33)	119.70 (38.84–368.88)	Medium
	ICD-10 I09.9, I11.0, I13.0, I13.2, I25.5, I42.2, I42.5-I42.9, I43.x, I50.x, P29.0		80.0 (67.03–89.57)	97.8 (93.78–99.55)	93.6 (82.46–98.66)	92.5 (86.92–96.18)		36.80 (11.92–113.58)	0.20 (0.12–0.35)	180.00 (48.03–674.60)	
**Henderson** [46], 2006	ICD-10 I50	1998–1999	87.0 4 (83.13–90.17)	99.44 (99.22–99.60)	89.89 (86.22–92.69)	99.26 (99.02–99.45)	0.88	155.87 (112.77–215.44)	0.13 (0.10–0.17)	1195.7 (769.3–1858.4)	High
		2000–2001	86.25 (79.71–91.00)	99.71 (99.55–99.81)	86.25 (79.71–91.00)	99.71 (99.55–99.81)	0.86	292.90 (192.10–446.59)	0.14 (0.09–0.20)	2123.9 (1148.9-3926.4)	
**Alqaisi** c [45], 2009	ICD-9 428.xx, 398.91, 402.01, 402.11, 402.91				65.00 (60.08–69.63)						High
**Chen** [47], 2009	ICD-10 I09.9, I11.0, I13.0, I13.2, I25.5, I42.0, I42.5- I42.9, I43.x, I50.x, P29.0	1^st^–16^th^ diagnostic positions	68.56 (63.24–73.45)	99.32 (98.98–99.55)	90.16 (85.65–93.41)	97.20 (96.61–97.70)		100.76 (67.72–149.92)	0.32 (0.27–0.37)	318.33 (201.73–502.32)	Medium
		kappa					0.76				
		prevalence-adjusted, bias-adjusted kappa					0.94				
**Heisler** [52], 2009	ICD-9 428, 428.0, 428.1, 428.9		0.00 (0.00–80.21)	100.00 (99.32–100.0)	n/a (0/0)	99.72 (98.87–99.95)					Low
**Merry** [54], 2009	ICD-9 428		43.04 (35.27–51.15)	99.92 (99.86–99.95)	80.00 (69.64–87.60)	99.57 (99.45–99.64)		530.43 (319.19–881.47)	0.57 (0.50–0.65)	930.44 (525.99–1645.9)	High

a Validation was conducted amongst a coronary care unit population.

b Thirty-eight algorithms were evaluated in this study; the first parameter we selected achieved the highest sensitivity with specificity ≥90%, the second was chosen for comparison purposes.

c Sixteen algorithms were evaluated in this study; the validity of the primary algorithm is reported here 95% CI = 95% confidence interval; DOR = diagnostic odds ratio; HF = heart failure; LR+  =  positive likelihood ratio; LR-  =  negative likelihood ratio; LVEF = left ventricular ejection fraction; MI = myocardial infarction; NPV = negative predictive value; PPV = positive predictive value.

The Framingham criteria were used in three studies, with the PPV's reported as 65% [45], 82% [42], and 94% [56]. One of these studies [56] used both the Framingham and Carlson criteria, and found higher accuracy with the Framingham (PPV = 94%) than with the Carlson (PPV = 89%). The European Society of Cardiology criteria were used in two studies; the PPV for definite HF was 82% in one [34] but just 65% in the other [17]. Sex-stratified statistics were provided by two studies; one [56] reported a slightly higher PPV for ICD-9 428 in females, and the other [43] found that the sensitivity of ICD-9 428 was significantly better in females than males (66% vs. 61%). In that study, the sensitivity was also significantly better amongst Mexican Americans than Non-Hispanic whites (66% vs. 59%) [43].

The studies in **Table 2** are ordered chronologically by publication year for the purpose of identifying any secular trends in the validity of HF codes. The nine-earliest studies included in this review were published between 1999 and 2004, with the ten remaining studies published from 2005 to 2009. However, no secular trends were observed for any of the validation statistics. Eleven (58%) of the studies included in this review were rated as high quality and seven (37%) as medium quality. Sensitivity ranged from 43% to 87% amongst the high quality studies, and from 58% to 87% amongst the medium quality. The PPV's for these two categories were also similar, ranging from 36% to 99% amongst the high quality studies, and from 34% to 100% amongst the medium quality. Any geographic comparisons were limited by the fact that 15 of the 19 studies were conducted in North America. The only difference observed was that the sensitivity values tended to be higher amongst the seven US studies than the five Canadian ones.

### Meta-Analysis

**Figure 2:**
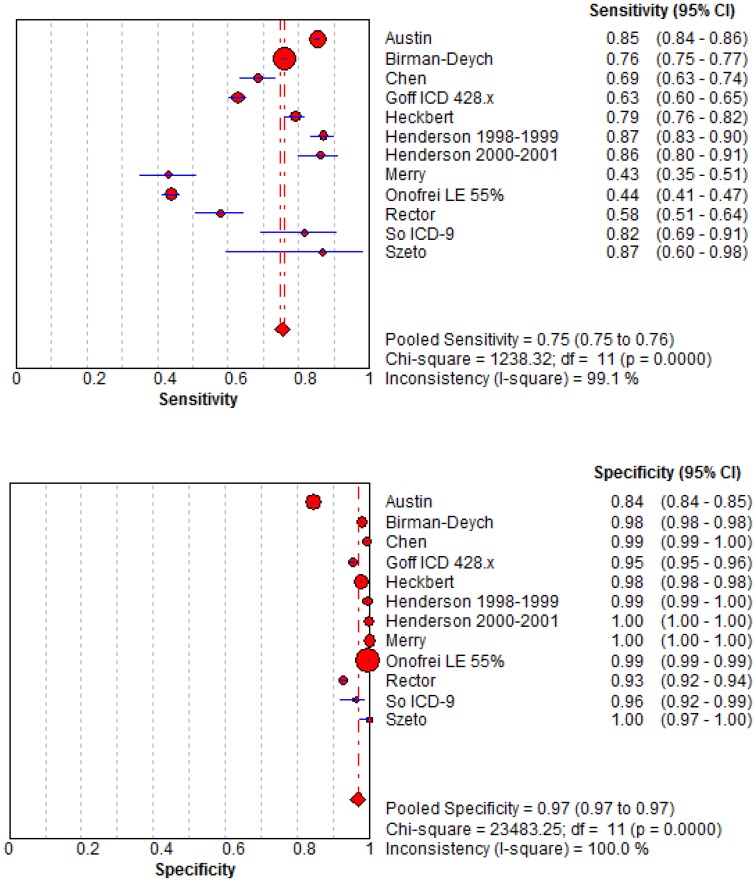
Forest Plots of Sensitivities and Specificities of Heart Failure Codes as Reported by Included Studies. 95% CI = 95% confidence interval; DF = degrees of freedom.

**Figure 3:**
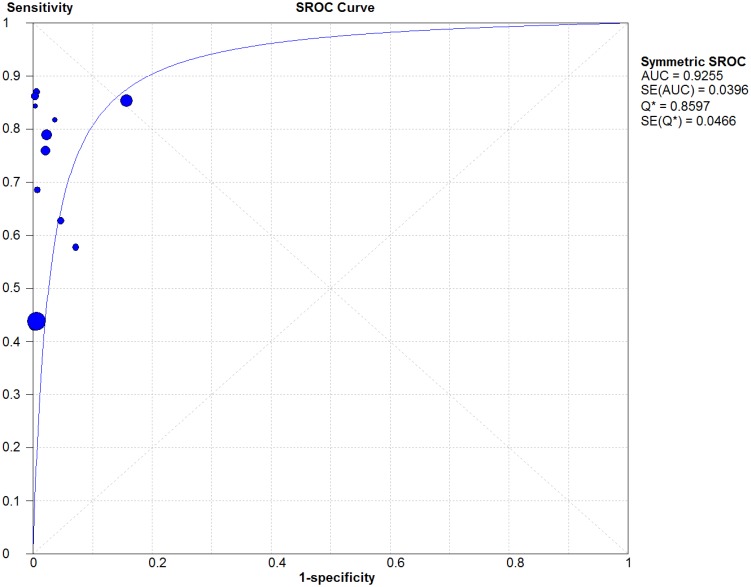
Receiver Operating Characteristic Curve Describing the Diagnostic Performance of Heart Failure Codes in Administrative Databases. AUC = Area Under the Curve; SROC = Summary Receiver Operating Characteristic; SE = standard error.

Included in the quantitative synthesis were the 11 articles from which raw data on sensitivity and specificity were available. Forest plots of the pooled sensitivity and specificity values are illustrated in **Figure 2**. A random-effects model was used since the χ^2^, Q*, and I^2^ statistics indicated there was a high level of heterogeneity between studies. The pooled sensitivity was 75.3% (95% CI: 74.7–75.9) and the pooled specificity was 96.8% (95% CI: 96.8–96.9). The summary LR+ was 51.9 (95% CI: 20.5–131.6) and the summary LR- was 0.27 (95% CI: 0.20–0.37), giving a summary DOR of 186.5 (95% CI: 96.8–359.22). The summary ROC curve is illustrated in **Figure 3**, wherein the AUC was 0.93 (SE 0.0396) and the Q* was 0.86 (SE 0.0466).

Results of the jackknife sensitivity analysis, wherein the pooled estimates were re-calculated after the removal of one study at a time, are shown in **Table 3**. The resulting pooled sensitivity estimates ranged mainly from 74% to 76%, and the specificities from 96.6% to 96.9%. The two most influential studies were those by Onofrei *et al* (sensitivity = 77.5% and specificity = 93.3% after its removal) and Austin *et al* (sensitivity = 71.8% and specificity = 99.1% after its removal).

**Table 3:** Results of Sensitivity Analysis Using a Jackknife Approach.

Omitted Study: First Author, Year of Publication	Records Evaluated (N)	Pooled Sensitivity (95% CI)	Pooled Specificity (95% CI)	Pooled LR+ (95% CI)	Pooled LR- (95% CI)	Pooled Diagnostic OR (95% CI)
**All studies included**	371,055	75.3 (74.7–75.9)	96.8 (96.8–96.9)	51.9 (20.5–131.6)	0.27 (0.20–0.37)	186.5 (96.8–359.22)
**Austin** [53], 2002	58,816	71.8 (71.1–72.5)	99.1 (99.1–99.1)	62.1 (35.2–109.6)	0.28 (0.21–0.38)	220.2 (119.1–407.3)
**Birman-Deych** [15], 2005	23,657	74.6 (73.7–75.4)	96.8 (98.7–96.9)	53.4 (21.2–134.7)	0.27 (0.18–0.40)	193.3 (90.0–415.2)
**Chen ** [47], 2009	4,008	75.4 (74.8–76.0)	96.8 (96.8–96.9)	48.8 (18.6–128.1)	0.26 (0.19–0.37)	176.9 (89.4–350.0)
**Goff** [43], 2000	5,083	76.2 (75.6–76.8)	96.9 (96.8–96.9)	59.2 (21.5–162.9)	0.26 (0.18–0.37)	221.9 (108.3–454.7)
**Heckbert** [55], 2004	34,016	75.2 (74.6–75.8)	96.8 (96.7–96.8)	54.5 (18.3–162.3)	0.28 (0.20–0.38)	189.9 (93.8–384.6)
**Henderson** [46], 2006: 1998–1999	7,004	75.1 (74.5–75.7)	96.8 (96.7–96.8)	46.7 (17.9–121.8)	0.29 (0.21–0.40)	154.4 (80.5–296.0)
**Henderson** [46], 2006: 2000–2001	7,631	75.2 (74.6–75.8)	96.8 (96.7–96.8)	44.1 (16.9–115.2)	0.29 (0.21–0.39)	147.8 (76.6–285.3)
**Merry** [54], 2009	21,110	75.6 (75.0–76.1)	96.6 (96.6–96.7)	41.8 (16.0–109.2)	0.25 (0.18–0.34)	159.7 (81.8–311.6)
**Onofrei** [44], 2004	205,756	77.5 (76.9–78.1)	93.2 (93.0–93.3)	49.8 (19.0–130.0)	0.25 (0.20–0.32)	193.56 (92.4–405.3)
**Rector** [49], 2004	3,633	75.5 (74.9–76.1)	96.9 (96.8–96.9)	62.2 (22.7–170.8)	0.26 (0.18–0.35)	235.5 (118.8–467.1)
**So** [48], 2006	193	75.3 (74.7–75.9)	96.8 (96.8–96.9)	55.9 (21.2–147.6)	0.28 (0.20–0.38)	193.1 (97.6–381.9)
**Szeto** [50], 2002	148	75.3 (74.7–75.9)	96.8 (96.8–96.9)	48.1 (18.6–124.9)	0.28 (0.20–0.38)	174.9 (89.9–340.3)

95% CI = 95% confidence interval; LR+  =  positive likelihood ratio; LR-  =  negative likelihood ratio; OR = odds ratio.

## Discussion

To our knowledge this is the first systematic review and meta-analysis on the validity of HF diagnoses in administrative data. Findings from this review suggest that the sensitivity of these codes is suboptimal, as sensitivity was ≤69% in 8 of the 14 studies reporting this statistic. However, the specificity and PPV of these codes appears much better: specificity was at least 95% in the 13 studies where this statistic was reported, and, in the majority of studies, the PPV was at least 87%. Further support was provided by the results of the meta-analysis, as the pooled specificity of HF codes was 97%, and the pooled LR+ was 52. This means an individual coded for HF is fifty-two-times more likely to actually have HF than someone not coded. However, the pooled sensitivity was modest, at just 75%, and the summary LR- value of 0.27 suggests that the absence of an HF code can rule out the diagnosis of HF only moderately.

The PPV's and NPV's amongst the studies included in this review were generally good, being at least 87% in the majority of studies reporting these statistics. A recently-published qualitative review of the validity of HF codes in North American databases also found the PPV to be generally high (>90% in most) [57]. However, it must be kept in mind that PPV and NPV are both dependent on the prevalence of the condition in the study population [35], and will be lower for rare conditions than for common conditions. This is important for HF because this condition differentially affects older individuals: for example, HF is reported to affect approximately 7.8% of US males aged 60–79 years, but only 1.5% of US males aged 40–59 years [2]. A higher baseline risk of HF in the study population may explain why several studies included in this review reported exceptionally high PPV's. For example, in the study by Szeto *et al* [50], which was conducted amongst a cohort of patients attending a Veteran's Affairs clinic, the prevalence of HF was 10%, and the PPV was 100%. So *et al* [48] examined the charts of patients hospitalized for MI, amongst whom the prevalence of HF was 29%, and the PPV in that study was 94%. Similarly, the prevalence of HF was 47% amongst the atrial fibrillation cohort studied by Birman-Deych *et al* [15], and the PPV was 97%. Consequently, if the exclusion of false-positive HF cases is of upmost priority for a particular study, the age and disease history of the study population must be taken into account when evaluating how accurately these codes will identify true HF cases.

Findings from our review suggest that administrative data codes are less-than-optimal for capturing HF cases, and this is consistent with another qualitative review of the validity of HF codes in which the sensitivity of HF diagnoses was highly-variable [58]. Instead of HF itself, some authors have suggested there is a tendency to list the underlying cause of the HF (such as MI or atrial fibrillation [17]), or another cardiac condition [53], in the primary position of the hospital discharge summary. Moreover, hospital coders generally report active conditions [51] such as MI, but may leave out chronic conditions such as HF if they were deemed not to have impacted the treatments provided in hospital or length-of-stay [47], [58]. Similarly, the study by Birman-Deych *et al*, where sensitivity for HF increased with disease severity (from 80% for mild cases to 94% for severe [15]), suggested that severe cases of HF may be recorded more often in administrative databases than mild ones. Thus, to maximize the capture of HF cases, authors are advised to broaden their search parameters by examining all diagnostic positions of the hospital record, considering the inclusion of more HF-related codes (other than ICD-9 428 or ICD-10 I50) in the search algorithm, and, where available, searching for HF cases in both hospitalization and outpatient databases.

### Sources of Administrative Data

While the improvements were not substantial, some studies we reviewed suggested HF cases could be identified more accurately if algorithms combining hospital codes with prescription data were applied. For example, Rector *et al* [49] tested several algorithms to identify HF, some of which incorporated prescription claims for an angiotensin converting enzyme (ACE) inhibitor, angiotensin-II receptor antagonist, loop diuretic, or digoxin. When using an algorithm that required a healthcare encounter and HF-related prescription, the specificity was high (92%), though the sensitivity was modest (53%) [49]. While further research is needed in this area, findings from that study also suggested that HF cases could be identified from prescription data alone, as the specificity of an algorithm that included a single HF-related prescription, but no healthcare encounters, was 78% [49]. This occurred despite the fact that many medications used in the treatment of HF are also used to treat other conditions [49]. Hence, prescription medication data could be used to validate HF cases first identified from hospital or outpatient data, or used alone to identify HF cases in a sensitivity analysis. Requiring that cases be dispensed a combination of medications - for example, each of a diuretic, ACE inhibitor or angiotensin-II receptor antagonist, and beta blocker - may improve specificity. At this time the prescription databases in many countries only include government-subsidized prescriptions, limiting the potential of this data source for identifying cases. Thus, prescription medication data should only be used if the database contains records on all community-dispensed prescriptions, regardless of payer, or at least all prescriptions dispensed to senior citizens.

Laboratory databases may also be a useful source for identifying HF. Specifically, levels of B-type natriuretic peptide(BNP) are often elevated in patients with left ventricular HF [6], so individuals with high BNP values could be identified as HF cases. One study in this review, by Alqaisi *et al* [45], compared the accuracy of different algorithms for identifying HF, some of which included BNP levels, and the highest-sensitivity algorithm in that study was ≥2 outpatient encounters for HF, or ≥1 hospitalizations for HF, or a BNP level of ≥200 pg/ml. That algorithm achieved a sensitivity of 76% and a specificity of 75%. BNP levels can be elevated in conditions other than HF, such as pulmonary embolism and chronic obstructive pulmonary disease [6], which may limit the specificity of BNP levels for identifying HF. However, additional findings from the Alqaisi *et al* study [45], where the specificities of BNP levels of ≥100 pg/ml, 200 pg/ml, and 500 pg/ml (without considering any diagnoses from healthcare encounters) were 76%, 88%, and 95%, respectively, suggest this test is reasonably specific for HF. Any potential increases in sensitivity will be limited by the fact that BNP levels tend to be elevated more in HF patients with left systolic dysfunction than diastolic dysfunction [6]. Another caveat is that BNP is less sensitive a test in non-acute HF [5]. Where laboratory data are available, we suggest researchers incorporate BNP levels into their case definition and, in a sensitivity analysis, compare the HF cases identified with- and without BNP levels.

### Reference Standards

Findings from our meta-analysis suggested a high degree of heterogeneity amongst the included studies; thus, a random-effects model was used to produce the summary measures. Part of this heterogeneity can be attributed to differences in the characteristics of the study populations. Some studies were community-based or conducted on a general hospitalized population while others were conducted on select populations (i.e. elderly people or those with a history of MI) in whom HF is more prevalent. Variations in the size of the study population may also have contributed, as there were changes, though not substantial, in the pooled sensitivity and specificity estimates after each of the two largest studies (n = 205,755 and n = 58,816) were removed.

More importantly, there was much heterogeneity in the reference standards used by different studies. This was not surprising as there is no single accepted gold standard for the diagnosis of HF, and a definitive diagnosis of HF is often difficult [53], [58], especially in elderly patients with multiple complications [34]. The reference standards used included patient self-report, chart reviews by clinicians and non-clinicians, two distinct disease registries, and the application of several sets of standard diagnostic criteria including the Framingham, Carlson, and European Society of Cardiology (ESC). One study included in our review, by Onofrei *et al* [44], reported both low sensitivity (44%) and low PPV (36%) for HF codes, which could be explained, in part, by their choice of reference standard. It consisted of a single measurement, left ventricular ejection fraction (LVEF) of either ≤55% or ≤40%. The LVEF is not typically used for HF diagnosis, but instead for classifying HF patients with left ventricular systolic or diastolic dysfunction [59]. Although the thresholds vary, an LVEF below 40% or 50% is usually indicative of systolic dysfunction, while higher LVEF values in HF patients are usually indicative of diastolic dysfunction but preserved systolic function [59]. It is possible that some of the false-positive cases in that study (coded for HF but whose LVEF measurement did not fall below the thresholds) exhibited other signs and symptoms that would fulfill the criteria for HF under the less-restrictive Framingham, Carlson, or ESC definitions. Thus, this choice of reference standard may have attenuated the PPV.

Furthermore, although the Framingham and Carlson criteria have been shown to be 100% sensitive to cases of definite HF [60], especially severe cases [61], the Framingham criteria are considered by some to be insensitive for detecting early HF [62], [63]. Therefore, the application of standard diagnostic criteria may also attenuate the PPV. In this review, we did observe a trend towards greater PPV (80-100%) when simply a physician's written confirmation of HF diagnosis or other notation in the medical chart was used as a gold standard [15], [42], [50]. In fact, Roger *et al* [42] compared two gold standards, physician diagnosis (as written in the chart) and the Framingham criteria, and found that the PPV from physician diagnosis was higher compared with the Framingham criteria (90% vs. 82%). Although the physician diagnosis may be more subjective, it may better reflect the diagnoses made in day-to-day clinical practice and thus be more meaningful to health researchers.

In addition to prescription medication and laboratory data, a third resource that could be used in conjunction with conventional administrative (billing) data to capture more HF cases is the electronic medical record (EMR), or electronic health record (EHR). The EMR or EHR is a digital file used by healthcare providers for patient care [64]. Though some authors use EMR when referring to the digital file maintained by a single practitioner, and EHR when referring to a digital file containing inpatient and outpatient data from multiple practitioners, for simplicity we will employ a single term, EMR, in this discussion. The materials available in the EMR can vary, but generally include clinical notes (similar to those recorded in a paper medical chart), prescription records, and laboratory and radiology reports [64]. With access to EMRs, researchers can identify HF cases by searching for an ICD code for HF amongst the patient's Problem List, a list maintained by the practitioner of all current and active diagnoses. In addition, researchers can also search for the term ‘heart failure’ amongst the entire free-text areas of the clinical notes, laboratory and radiological reports, and any correspondence from specialists. For example, the Mayo Clinic has used a natural language processing (NLP) algorithm containing the terms ‘cardiomyopathy’, ‘heart failure’, ‘congestive heart failure’, ‘pulmonary edema’, ‘decompensated heart failure’, ‘volume overload’ and ‘fluid overload’, along with 426 synonyms for these terms [65]. A potential case is eliminated if a negative term (such as ‘no’ or ‘unlikely’), or sometimes even a speculative term (such as ‘rule-out’ or ‘suspected’) is found within close proximity of the HF term [65], [66]. For chronic conditions like HF that may be superseded by other diagnoses on reimbursement claims, this EMR-based search strategy may be more sensitive than relying on the diagnostic codes in claims-based administrative databases, and may aid in capturing milder cases.

However, the EMR also has some limitations, many of which stem from the fact that, similar to most administrative databases, the EMR was not established for research purposes [64]. For example, when searching the free-text notes and reports, computer programs may have difficulty processing whether ambiguous phrases like “cannot be ruled out” [67] correspond to a positive case. In addition, while the information contained in administrative databases is already de-identified, the EMR does contain personally-identifying information. US law stipulates that, unless each patient provides consent [68], researchers cannot use data collected from the EMR without it undergoing a de-identification process [69]. Thus, it may be costly and time-consuming for researchers to access this data, especially as many hospitals do not use de-identification tools at present [68]. A lack of standardization across EMR systems [64] and challenges in linking EMRs from different hospitals or provider networks [70] may also limit the use of EMR. Finally, just as with ICD codes, the validity of the HF cases identified from the free-text areas of the EMR must be assessed before EMRs can be used for HF research. While the results of some validation studies [65], [67] have been promising, more validation studies conducted in different settings, and using different EMR platforms, are needed to confirm these findings.

### Limitations

We acknowledge some limitations to our systematic review. There is the potential for a language bias as we could not consider articles whose full-texts were not available in English; articles published after the conclusion of our search period (February 2011) could not be considered either. Another potential limitation stems from the fact that, even though our database searches were conducted by an experienced librarian, administrative databases are not well catalogued in MEDLINE and EMBASE (e.g. no MeSH term pertaining to “administrative database”). Although most of the included studies were located through database searches, our subsequent hand search turned up several more relevant articles, most of which had not been indexed under terms relating to Administrative Data or Validation. As a result, despite our extensive hand search, we may have missed some relevant articles if they were not indexed in MEDLINE or EMBASE under a term relating to administrative data or validation. Our findings are also subject to publication bias, wherein reports of HF codes having poor validity may have been differentially withheld from publication. However, given the number of reports we located where the sensitivity of HF codes was suboptimal, we feel this is unlikely

### Recommendations

After qualitative and quantitative analysis of the evidence, we conclude that the HF codes that do appear in administrative databases are highly predictive of true HF cases. At the same time, administrative databases fail to capture a non-negligible number of true cases, perhaps 25% to 30% of all diagnoses, and may differentially capture the most severe cases. Based on current evidence, we recommend several strategies for increasing the capture of HF cases in administrative data:

- Hospitalizations with HF in the primary position are decreasing, while those with HF in secondary positions are increasing [26], [27]. Thus, researchers should search amongst all available diagnostic positions in hospitalization data for HF codes.
- With many HF patients treated exclusively on an outpatient basis, data from both inpatient and outpatient encounters should be searched.
- Where available, researchers should supplement their data with searches of laboratory databases (specifically BNP values) and/or prescription medication data.
- Searching the free-text areas of the EMR for mentions of ‘heart failure’ and related terms, as well as the Problem List, should help identify cases whose HF diagnosis has not been recorded on an inpatient or outpatient billing record. This may particularly aid in the capture of mild HF.

### Conclusions

The chronic and syndromic nature of HF creates difficulties for researchers studying this condition at the population level. To guide their efforts, we conducted a systematic review and meta-analysis of articles reporting on the validity of HF diagnoses in administrative data. Our findings suggest that, although the HF diagnoses identified using administrative data frequently correspond to true HF cases, this data source may not capture all cases. Administrative databases are increasingly being used to study long-term patient outcomes and disease burden; thus, to maximize the sensitivity of these data sources for all conditions, physicians and hospital coders are encouraged to record diagnoses of all comorbidities that may have contributed to a given healthcare encounter. In the meantime, the use of broader case definitions, potentially in combination with prescription medication and laboratory data, and searches of electronic medical records, may increase the sensitivity of this data source for HF, and in turn, its application in population-based health outcomes and economics research.

## Supporting Information

Table S1 **Item-by-Item QUADAS Breakdown for Each Study.**
(DOC)

Text S1 **MEDLINE Search Strategy.**
(DOCX)

Text S2 **EMBASE Search Strategy.**
(DOCX)

Text S3 **Data Collection Form.**
(DOC)

Checklist S1 **PRISMA Checklist.**
(DOC)

Checklist S2 **MOOSE Checklist.**
(DOCX)
