# Supplementary material for: Validity of Heart Failure Diagnoses in Administrative Databases: A Systematic Review and Meta-Analysis
Source: PLoS One. 2014 Aug 15;9(8):e104519. doi: 10.1371/journal.pone.0104519 (PMC4134216; doi:10.1371/journal.pone.0104519)
Supplement: Table S1 — Item-by-Item QUADAS Breakdown for Each Study. (DOC) [file pone.0104519.s001.doc]

**Table S1: Item-by-Item QUADAS Breakdown for Each Study**

| **QUADAS Question no.** | **Alqaisi**[45] | **Austin**[53] | **Birman-Deych**[15] | **Chen**[47] | **Goff**[43] | **Heckbert**[55] | **Heisler**[52] | **Henderson**[46] | **Humphries[**51] | **Ingelsson**[34] |
| --- | --- | --- | --- | --- | --- | --- | --- | --- | --- | --- |
| **1** | Y | Y | N | Y | Y | N | N | Y | N | N |
| **2** | Y | Y | Y | Y | Y | Y | Y | Y | Y | Y |
| **3** | Y | Y | N | U | U | Y | U | U | N | Y |
| **4** | NA | NA | NA | NA | NA | NA | NA | NA | NA | NA |
| **5** | Y | Y | Y | Y | Y | Y | Y | Y | Y | Y |
| **6** | Y | Y | Y | Y | Y | Y | Y | Y | Y | Y |
| **7** | N | Y | Y | Y | Y | Y | Y | Y | Y | N |
| **8** | Y | Y | Y | Y | Y | Y | Y | Y | Y | Y |
| **9** | Y | Y | U | N | Y | N | N | Y | Y | Y |
| **10** | Y | Y | Y | Y | Y | Y | Y | Y | Y | Y |
| **11** | U | Y | Y | U | Y | U | U | U | U | U |
| **12** | Y | Y | Y | Y | Y | Y | Y | Y | Y | Y |
| **13** | N | Y | Y | NA | N | U | Y | N | N | Y |
| **14** | NA | NA | NA | NA | NA | NA | NA | NA | NA | NA |
| **Overall Quality Assessment** | High | High | Medium | Medium | High | High | Low | High | Medium | High |

Y=Yes; N=No; U=Unclear; NA=Not Applicable

| **QUADAS Question no.** | **Khand**[17] | **Lee**[56] | **Levy**[16] | **Merry**[54] | **Onofrei**[44] | **Rector**[49] | **Roger**[42] | **So**[48] | **Szeto**[50] |
| --- | --- | --- | --- | --- | --- | --- | --- | --- | --- |
| **1** | Y | Y | N | Y | Y | N | Y | N | N |
| **2** | Y | Y | Y | Y | Y | Y | Y | Y | Y |
| **3** | Y | Y | N | Y | Y | N | Y | U | N |
| **4** | NA | NA | NA | NA | NA | NA | NA | NA | NA |
| **5** | Y | Y | Y | Y | Y | Y | Y | Y | Y |
| **6** | Y | Y | Y | Y | Y | Y | Y | Y | Y |
| **7** | Y | N | N | Y | Y | Y | N | Y | Y |
| **8** | Y | Y | Y | Y | Y | Y | Y | Y | Y |
| **9** | Y | Y | Y | Y | Y | Y | Y | N | Y |
| **10** | Y | Y | Y | Y | Y | Y | Y | Y | Y |
| **11** | U | U | NA | Y | U | Y | U | U | U |
| **12** | Y | Y | Y | Y | Y | NA | Y | Y | Y |
| **13** | Y | Y | NA | U | N | Y | Y | NA | N |
| **14** | NA | NA | NA | NA | NA | NA | NA | NA | NA |
| **Overall Quality Assessment** | High | High | Medium | High | High | Medium | High | Medium | Medium |

Y=Yes; N=No; U=Unclear; NA=Not Applicable

*QUADAS questions are displayed in Text S3
