# Supplementary material for: Validity of Heart Failure Diagnoses in Administrative Databases: A Systematic Review and Meta-Analysis
Source: PLoS One. 2014 Aug 15;9(8):e104519. doi: 10.1371/journal.pone.0104519 (PMC4134216; doi:10.1371/journal.pone.0104519)
Supplement: Checklist S2 — MOOSE Checklist. (DOCX) [file pone.0104519.s006.docx]

| **Criteria**  **Checklist S2: MOOSE Checklist** | **Brief description of how the criteria were handled in the meta-analysis** |
| --- | --- |
| **Reporting of background should include** | |
| Problem definition | Administrative databases are increasingly being used to study heart failure (HF). Investigations into the validity of HF diagnoses in administrative data vary greatly in terms of the populations under study and reference standards used. To synthesize this evidence on a larger scale, we have undertaken a systematic review and meta-analysis of studies reporting on the validity of HF codes in administrative data. |
| Hypothesis statement | NA |
| Description of study outcomes | Diagnosis of HF made in accordance with a reference standard. |
| Type of exposure or intervention used | International Classification of Diseases (ICD) diagnostic code for HF (ICD-8 or ICD-9 428, or ICD-10 I50) recorded in an administrative database. |
| Type of study designs used | We searched for studies (a) Using administrative data to identify HF; or (b) Evaluating the validity of HF codes in administrative data; and (c) Reporting validation statistics (sensitivity, specificity, positive predictive value, negative predictive value, or Kappa scores) for HF, or data sufficient for their calculation. There was no *a priori* restriction placed on the study design; however, due to the nature of our research question, most of the included studies were observational and retrospective. |
| Study population | There was no restriction on study population. |
| **Reporting of search strategy should include** | |
| Qualifications of searchers | The database search was conducted by an experienced librarian, Mary-Doug Wright, B.Sc., M.L.S. The handsearch was conducted by the authors, whose credentials are reported. |
| Search strategy, including time period included in the synthesis and keywords | MEDLINE 1946 to November 2010  EMBASE 1974 to November 2010  Our complete search strategies are available in supplementary materials Text S1 and Text S2. |
| Databases and registries searched | MEDLINE and EMBASE |
| Search software used, name and version, including special features used | The database searches were conducted using the OvidSP platform. The search features included Mesh/Subject Heading explosion, truncation, specific field searching (e.g., Mesh/SH, title, abstract), Boolean logic, and duplicates removal. EndNote X4 was used for bibliographic management of downloaded records from the Ovid databases |
| Use of hand searching | To find additional articles, the authors hand-searched the reference lists of the key articles located through the database search. The Cited-By tools in PubMed and Google Scholar were also used to find relevant articles that had cited the articles located through the database search. |
| List of citations located and those excluded, including justifications | The citations for all included articles are listed in the References section. |
| Method of addressing articles published in languages other than English | No language restrictions were put on the initial database searches. We located six records whose full-text was not published in English. These records were excluded because we could only include articles whose full-text was published in English. |
| Method of handling abstracts and unpublished studies | Of the six abstracts located where no full-text article had been published in any language, two reported on the validity of HF diagnoses. The authors of these abstracts were not contacted. |
| Description of any contact with authors | We did not contact the authors of any studies identified in the literature search. |
| **Reporting of methods should include** | |
| Description of relevance or appropriateness of studies assembled for assessing the hypothesis to be tested | The inclusion criteria are detailed under the Literature Search and Inclusion Criteria subheadings of the Methods section. |
| Rationale for the selection and coding of data | As detailed in the Data Extraction subheading of the Methods section, we abstracted the ICD codes and validation statistics as reported by each study, along with raw data on the number of true- and false- positive cases, and true- and false- negative cases. Data on factors known to influence the validity of diagnostic codes in administrative data – including the study population and setting, administrative data source, validation method, and reference standard – were also abstracted. |
| Documentation of how data were classified and coded | Data were independently abstracted by each reviewer and then compared to identify and correct errors and discrepancies. Data were abstracted using a standardized collection form, a copy of which is provided in Text S3. |
| Assessment of confounding | Subgroup analyses were conducted according to the time period of publication, geographic region of publication, and level of study quality (High, Medium, or Low). |
| Assessment of study quality, including blinding of quality assessors; stratification or regression on possible predictors of study results | All studies were evaluated for quality using the Quality Assessment of Diagnostic Accuracy Studies (QUADAS) tool, a 14-item evidence-based quality assessment tool used in systematic reviews of diagnostic accuracy studies that incorporates aspects of bias and applicability. These items were used to qualitatively assess each study as High, Medium, or Low quality. We report on the quality assessments under the Study Characteristics subheading of the Results section, and in Table 2. |
| Assessment of heterogeneity | Heterogeneity of studies was assessed using the χ^2^ statistic, Cochran’s Q, and the *I*^2^ statistic, which is a measure of the variation in study findings that is due to heterogeneity as opposed to chance. |
| Description of statistical methods in sufficient detail to be replicated | Under the Statistical Analysis subheading of the Methods section is a detailed description of the validation statistics that were analyzed, and how these were calculated from the raw data reported by individual studies. Details of how the meta-analysis was conducted are provided under the Meta-Analysis subheading of the Methods section. |
| Provision of appropriate tables and graphics | Figure 1 is a flow diagram (constructed in accordance with the Preferred Reporting Items for Systematic Reviews and Meta-Analyses (PRISMA) reporting guidelines) illustrating the study search and selection process. The characteristics of each included study are provided in Table 1, and the item-by-item quality scores for each included study are provided in Table S1. The validation statistics reported by each study are provided in Table 2. Results of the jackknife sensitivity analysis are provided in Table 3. Figure 2a is a forest plot of the sensitivity values reported by each study that was included in the meta-analysis. Figure 2b is a forest plot of the specificity values reported by each study included in the meta-analysis. Figure 3 is the summary receiver operating characteristic curve. |
| **Reporting of results should include** | |
| Graphic summarizing individual study estimates and overall estimate | Table 2  Figure 2 |
| Table giving descriptive information for each study included | Table 1 |
| Results of sensitivity testing | Results of the jackknife sensitivity analysis are reported under the Meta-Analysis subheading of the Results section, and in Table 3 |
| Indication of statistical uncertainty of findings | The 95% confidence intervals were reported alongside with all summary estimates. |
| **Reporting of discussion should include** | |
| Quantitative assessment of bias | As explained under the Meta-Analysis subheading of the Methods section, we did not evaluate the impact of publication bias because the common statistical tests used to assess publication bias have been shown to be misleading for meta-analyses of test accuracy. We do acknowledge in the Discussion section how our findings are subject to publication bias. |
| Justification for exclusion | In the Discussion section we acknowledge we were unable to include any articles if the full-text was not published in English, or if they were published after the conclusion of our search period. |
| Assessment of quality of included studies | All included studies were assessed for quality using the Quality Assessment of Diagnostic Accuracy Studies (QUADAS) tool. This is a 14-item evidence-based quality assessment tool used in systematic reviews of diagnostic accuracy studies. Each item, phrased as a question, addresses one or more aspects of bias or applicability; however, there is no overall score. These items were used to qualitatively assess each study as High, Medium, or Low quality. Our quality assessments are reported under the Study Characteristics subheading of the Results section, and in Table 2. The validation statistics are also stratified by level of study quality. |
| **Reporting of conclusions should include** | |
| Consideration of alternative explanations for observed results | Positive predictive value (PPV) and negative predictive value (NPV) are dependent on the prevalence of the condition in the population and will be lower for rare conditions than for common conditions. Some studies in this review were conducted on general community-based or hospitalized populations while others were conducted on select populations, such as those with a history of myocardial infarction. In the Discussion section, we provide examples of studies in which HF was highly prevalent in the study population, and the PPV of HF diagnostic codes was especially high. Thus, studying populations where HF was more prevalent may have contributed to high PPVs reported by some studies in this review, and, in turn, the high PPV associated with HF diagnoses overall. |
| Generalization of the conclusions | We performed a systematic review and meta-analysis on the validity of diagnostic codes for HF in administrative data. Nineteen studies were included in the qualitative review and 11 in the quantitative synthesis. A high level of heterogeneity was observed amongst the studies, much of which we believe can be attributed to the variety of reference standards that were used. These include patient self-report, chart reviews by clinicians and non-clinicians, two distinct disease registries, and the application of several sets of standard diagnostic criteria. Findings from the systematic review suggest that the sensitivity of these codes is suboptimal (≤ 69% in 8 of the 14 studies reporting this statistic). The specificity, PPV, and NPV of these codes appear to be much better than the sensitivity (specificity was ≥ 95% in all 13 studies reporting this statistic, PPV was ≥ 87% in the majority of studies where this was reported, and NPV was ≥ 88% in all but two of 14 studies). Findings from the meta-analysis supported these results, as the pooled sensitivity of HF codes was 75% while the pooled specificity was 97%. Based on the evidence, we conclude that while approximately 25 to 30% of HF cases will not be captured in administrative data, the codes that are captured are highly predictive of true HF cases. |
| Guidelines for future research | Findings from some studies included in this review suggest the use of prescription medication data, either alone or in combination with hospitalization and outpatient data, could improve the identification of HF cases in administrative databases. Thus, we recommend more research be conducted in this area. |
| Disclosure of funding source | This study was funded in part by the Canadian Arthritis Network (http://www.arthritisnetwork.ca). |
